# Workplace-Sexual-Harassment Victimization and Employee Wellbeing Among LGBTQ+ and Non-LGBTQ+ Employees

**DOI:** 10.1177/08862605241285994

**Published:** 2024-10-08

**Authors:** Francisco Perales, Alice Campbell, Nicki Elkin

**Affiliations:** 1 The University of Queensland, Brisbane, Australia; 2 ACON Health, Sydney, Australia

**Keywords:** Australia, health, LGBTQ+, sexual harassment, wellbeing, workplaces

## Abstract

Workplace sexual harassment represents a critical risk to contemporary organizations, with evidence indicating that its prevalence is increasing. Research has consistently demonstrated that workplace-sexual-harassment victimization exerts negative impacts on employees’ health and wellbeing. However, no empirical studies have examined how these impacts vary by lesbian, gay, bisexual, trans, and queer (LGBTQ+) status. In this study, we leverage a unique survey dataset (2022 Australian Workplace Equality Index Employee Survey, n = 44,943) and random-intercept, multilevel regression models to estimate the relationships between workplace-sexual-harassment victimization and employee wellbeing. Expanding on earlier studies, we consider how these relationships vary between LGBTQ+ and other employees, across domains of employee wellbeing, and with the timing of sexual harassment. Our results reveal large, negative, and statistically significant impacts of sexual harassment on employee wellbeing. The impacts are comparatively larger for LGBTQ+ employees and recent harassment experiences, and manifest across all domains of employee wellbeing. These findings underscore the urgent need for holistic programs to combat workplace sexual harassment, and the importance of connecting these programs with diversity and inclusion initiatives.

Sexual harassment constitutes a critical social problem, and recent evidence suggests that its overall prevalence is on the rise (Australian Bureau of Statistics, 2021). Within the workplace, sexual harassment occurs when employees receive unwelcome sexual advances, requests for sexual favors, remarks with sexual connotations, or other unwelcome conduct with the intention of offending, humiliating, or intimidating (Australian Human Rights Commission [AHRC], 2022). Such behaviors may be typically performed by co-workers, superiors, and clients (AHRC, 2022). According to a recent study, 27% of employees in Australia—where the present study is based—report having ever experienced workplace sexual harassment, with 3% having been harassed within the past 12 months (Perales et al., 2024). For some subgroups, including women and lesbian, gay, bisexual, trans, and queer (LGBTQ+) people, rates can be twice as high (Perales et al., 2024). Similar estimates have been documented in studies conducted in other countries from the Global North, such as the United States and Germany (Edison Research, 2018; Federal Anti-Discrimination Agency, 2015).

Workplace sexual harassment can have substantial negative repercussions for organizations, dampening brand reputation, productivity, and talent attraction/retention, as well as incurring financial implications through settlements for sexual-harassment lawsuits (Au et al., 2024; McLaughlin et al., 2017; Shaw et al., 2018). In addition, its impacts on individual employees are well-documented. The present study is focused on employee wellbeing (sometimes referred to as “occupational” or “workplace” wellbeing): a multidimensional concept encompassing workplace-related outcomes such as productivity, attachment, engagement, and psychological wellbeing (Lyubomirsky, 2001; Page & Vella-Brodrick, 2009; Wijngaards et al., 2022; Zheng et al., 2015). In two landmark meta-analyses, Chan et al. (2008) and Sojo et al. (2016) identified that workplace-sexual-harassment victimization was negatively associated with multiple outcomes covering different domains of employee wellbeing. In particular, workplace-sexual-harassment victimization was related to decreases in organizational commitment, satisfaction with co-workers, satisfaction with supervisors, job performance and job satisfaction, and to increases in the risk of withdrawal from work and experiencing job stress (Chan et al., 2008; Sojo et al., 2016).

In addition, the harmful impacts of workplace sexual harassment on individuals are not restricted to the workplace. Rather they can have an “accumulative impact on more distal health outcomes that are not domain specific” (Sojo et al., 2016, p. 16). Such distal outcomes include measures of physical health (e.g., cardiovascular disease), mental health (e.g., anxiety and depression), life satisfaction, and health-risk behaviors (e.g., substance abuse)—see Chan et al. (2008) for a review. For example, recent studies have documented increases in hypertension (Lawn et al., 2022), depressive symptoms (Rugulies et al., 2020), alcohol-related morbidity and mortality (K. J. Blindow et al., 2023), and suicide (Magnusson Hanson et al., 2020) among individuals who have been sexually harassed at work. These broader physical and mental health sequelae of sexual harassment can recursively hamper employee wellbeing, increasing the risk of long-term sickness absence from work (K. Blindow et al., 2021) and exit from employment for health-related reasons (Sterud et al., 2023).

While these studies have paved the way for deeper understandings of the intra-individual consequences of workplace sexual harassment, important gaps in knowledge remain. First, most recent research in this space is restricted to a handful of countries—particularly, the United States and the Nordic countries (Friborg et al., 2017; Lawn et al., 2022; Magnusson Hanson et al., 2020; Rugulies et al., 2020; Sterud et al., 2023). The present study extends the evidence base to a new country context: Australia. In doing so, it contributes to establishing the generalizability of existing findings. Second, and most importantly, existing studies have rarely considered heterogeneity in the health-and-wellbeing effects of sexual-harassment experiences across population groups. This is an important omission, as some employee groups are more likely than others to experience sexual harassment at work and/or may experience greater vulnerabilities that could amplify its negative consequences (Chan et al., 2008; Hansen et al., 2020). As an exception, several studies have considered moderation by gender, finding no apparent difference in the effects of workplace sexual harassment on men versus women (Blindow et al., 2023; Magnusson Hanson et al., 2020; Rugulies et al., 2020). The present study delves into moderation by an important yet under-researched social location: whether or not employees identify as LGBTQ+. Hereon, we use this widespread acronym as a shorthand to refer to all individuals whose gender identity differs from their sex assigned at birth (e.g., trans and gender non-binary people) and/or whose sexual identity deviates from the heterosexual norm (e.g., gay/lesbian and bisexual people).

## Sexual Harassment and Employee Wellbeing Among LGBTQ+ Employees

Within heteronormative societies, LGBTQ+ individuals are afforded an underprivileged social status due to deeply entrenched societal stigma and discrimination (Meyer, 2003; van der Toorn et al., 2020). The ensuing power disparities between LGBTQ+ and other employees can explain the disproportionate rates of sexual-harassment victimization observed for LGBTQ+ employees (Konik & Cortina, 2008; Perales et al., 2024). Here, we argue that LGBTQ+ employees are not only overexposed to workplace sexual harassment, but also experience more deleterious consequences when this occurs. This theoretical expectation rests on four interrelated reasons.

First, as posited by the stress process model, the health impacts stemming from exposure to a new stressor are moderated by the presence of other stressors and the amount of earlier stress individuals have previously sustained (Pearlin, 1989, 2010; Pearlin et al., 1981). Greater levels of accumulated stress are associated with enhanced vulnerability when facing a new stressor—a process often referred to as stress proliferation (Pearlin et al., 1997). As posited by the minority stress theoretical perspective (Meyer, 2003; Perales & Todd, 2018), due to their sexual and/or gender diversity, LGBTQ+ people are exposed to unique stressors within and outside workplaces—including misgendering, bullying, ostracization, and limited legislative recognition (Donaghy & Perales, 2024; Galupo & Resnick, 2016; Resnick & Galupo, 2019). Hence, we expect the excess stress induced by becoming the target of sexual harassment to compound with higher existing stress levels among LGBTQ+ people, resulting in more pronounced health-and-wellbeing penalties.

Second, a substantial share of the population still holds negative attitudes about sexual and gender minorities (Hollekim & Anderssen, 2022; Kaufman & Compton, 2021; Perales & Campbell, 2018). As a result, on average, LGBTQ+ individuals tend to have more limited support networks—including family supports (Perales & Plage, 2020) and other supports (Perales & Todd, 2018). The stress process and other theoretical perspectives underscore the protective role of social support in the face of stressors (Pearlin, 1989, 2010; Pearlin et al., 1981). Consistent with this tenet, studies on workplace sexual harassment recognize the role of support sources in buffering against ensuing trauma (Nielsen et al., 2020). Thus, comparatively low levels of social support among LGBTQ+ employees experiencing workplace sexual harassment may result in more pronounced health-and-wellbeing penalties for this group.

Third, due to organizational- and institutional-level stigma and lack of preparedness to deal with diversity, LGBTQ+ employees can face discrimination during the hiring process (Grant et al., 2011; Pew Research Center, 2013) and have more complicated relationships with their employers (Cech & Rothwell, 2020; Lewis & Pitts, 2017). Low levels of employer engagement with diversity and inclusion practice may lead LGBTQ+ employees to distrust the system, as well as increasing their vulnerability to “institutional betrayal” (Smith & Freyd, 2014) upon reporting sexual harassment. Institutional betrayal occurs when an institution on which a victim/survivor is dependent acts in ways that worsen and perpetuate the trauma, distress, and harm experienced (Duffy et al., 2023). As for other marginalized groups (Duffy et al., 2023), preliminary evidence suggests that LGBTQ+ people may be more likely to experience institutional betrayal when sexual harassment occurs (Smidt et al., 2021; Smith et al., 2016). At the same time, greater difficulty to attain alternative, suitable employment may leave LGBTQ+ employees “trapped” in jobs within organizations where harassment took place (Dilmaghani & Robinson, 2024; Mills & Oswin, 2024). Together, institutional betrayal and an impaired ability to leave jobs where harassment occurred may exacerbate the negative wellbeing impacts of sexual harassment for LGBTQ+ employees.

Finally, the nature and triggers of victimization may be distinct and more harmful when workplace sexual harassment is directed at LGBTQ+ people. For this group, their sexuality diversity is both a core part of their sense of self and a source of deviance from traditional social scripts that can be deliberately invoked in acts of harassment (Konik & Cortina, 2008; Perales et al., 2024). For example, the literature documents instances of workplace sexual harassment where perpetrators ask gay men intrusive questions about their sexual roles, invite bisexual women to participate in group sex, or tell lesbian women that having sex with a man would “cure” them of their homosexuality (Trades Union Congress [TUC], 2019). These forms of harassment tied to individuals’ identity may exert more detrimental effects on health and wellbeing, reinforcing our expectation of larger penalties among LGBTQ+ employees.

Altogether, based on this literature, we expect employees who identify as LGBTQ+ to be more negatively affected by experiences of sexual harassment at work than other employees. In addition to subjecting this proposition to empirical scrutiny, the present study will explore two novel analytic avenues. First, it will compare the effects of sexual harassment on different domains of employee wellbeing (e.g., productivity, engagement, and sense of belonging; Wijngaards et al., 2022; Zheng et al., 2015), and how such effects may vary by employees’ LGBTQ+ status. This is an important endeavor, as it helps ascertain whether workplace sexual harassment bears uniform impacts across the board or, rather, it disproportionately affects certain domains. It can also help identify which domains contribute to any observed disparities by LGBTQ+ status. Second, this study will examine how the timing of sexual-harassment experiences moderates their health-and-wellbeing effects. As proposed in the stress process model, recent stressors should exert more deleterious effects on individuals’ health and wellbeing than distal ones (Pearlin, 1989, 2010; Pearlin et al., 1981). It follows that more recent sexual-harassment victimization should have more profound consequences on employees’ wellbeing. Whether or not the impact of recent and distal sexual-harassment experiences varies by LGBTQ+ status remains an open question that will be addressed empirically in our analyses. To our knowledge, no previous studies have tested these propositions.

## Data and Methods

### The 2022 Australian Workplace Equality Index Employee Survey

To accomplish our novel analytic aims, the present study leverages unique data from the Australian Workplace Equality Index (AWEI) Employee Survey, a large-scale, employer-employee, annual survey collected by ACON Health—Australia’s largest not-for-profit organization dedicated to LGBTQ+ health and wellbeing. The AWEI Employee Survey collects information on workplace experiences from employees of all genders and sexual orientations. Participating organizations are either members of ACON Health’s Pride Inclusions Programs, or organizations that choose to participate. All employees within these organizations are encouraged to complete the survey instrument. The AWEI Employee Survey has proven to be a valuable data source to inform academic research on the predictors of employee wellbeing, including inclusive language at work (Perales et al., 2022a, 2022b), diversity training and ally groups (Perales, 2022), and specific genders and sexualities (Donaghy & Perales, 2024). The present study utilizes data from the 2022 AWEI Employee Survey, the only annual installment to date to include a module on workplace sexual harassment. The survey was completed by 44,943 employees working in 182 organizations (including 9,806 employees who identified as LGBTQ+). The present study received ethical clearance by the authors’ institutional board (ID 2023/HE001965).

### Employee Wellbeing

The analytic outcome of interest is self-reported levels of employee wellbeing. To operationalize this, we derive a composite index following earlier studies (Donaghy & Perales, 2024; Perales, 2022; Perales et al., 2022a). The measure combines information on the different dimensions of employee wellbeing recognized in the literature (Lyubomirsky, 2001; Page & Vella-Brodrick, 2009; Wijngaards et al., 2022; Zheng et al., 2015), including workplace wellbeing—captured by feelings of productivity and engagement with one’s job, psychological wellbeing—captured by feelings of safety and belonging in the workplace, and subjective wellbeing—captured by feelings of being mentally well at work. Specifically, respondents were asked to rate their agreement with the following six statements: (a) “*I feel safe and included within my immediate team*,” (b) “*I feel mentally well at work*,” (c) “*I feel I can be myself at work*,” (d) “*I feel productive at work*,” (e) “*I feel engaged with the organization and my work*,” and (f) “*I feel a sense of belonging here*”. Respondents’ degree of agreement was captured on a Likert scale (1 = “*Strongly disagree*”; 5 = “*Strongly agree*”). The scores on the six items were first averaged and then combined into an index ranging from 0 (lowest wellbeing) to 100 (highest wellbeing). The latter was accomplished through the following linear transformation: index score = (average item score − 1) × 20. The resulting index exhibited optimal statistical properties. First, it featured a remarkable degree of internal consistency (Cronbach alpha = .92) and optimal item-rest correlations (*r* = .70–.84). Removing any one item did not increasing the Cronbach alpha score. Second, principal component analyses provided strong evidence of unidimensionality, with only one factor with an Eigenvalue above one (Eigenvalue = 3.99) explaining 72% of the variance. Further, all scale items were positively and highly correlated with this factor (*r* = .73–.87).

### Workplace-Sexual-Harassment Victimization

A key explanatory variable in the analyses is workplace-sexual-harassment victimization. The 2022 AWEI Employee Survey asks employees about their experiences of workplace sexual harassment through the question: “*Have you ever been sexually harassed at work?*”. To ensure appropriate comprehension by all survey respondents, the term “sexual harassment” was further contextualized as follows: “*Examples of sexual harassment include being the target of unwelcome/inappropriate physical contact, sexually explicit comments or gestures, receiving intrusive questions about your private life, and inappropriate advances or requests for sex.*” This way of asking about workplace sexual harassment mirrors the approach used in recent, major industry reports (AHRC, 2022; TUC, 2019). In answering this question, survey participants are asked to tick one of the following response options: “*Yes, within the past 12* *months*,” “*Yes, more than 12* *months ago*,” and “*No, never.*” In our analyses, we consider both (a) a binary measure of having ever experienced sexual harassment combining both “Yes” responses, as well as (b) a more nuanced measure that separates more and less recent sexual-harassment experiences.^1^

### LGBTQ+ Status and Sex Recorded at Birth

A second key explanatory variable is employees’ LGBTQ+ status. We operationalize this using the survey question “*Are you someone of diverse sexuality or diverse gender (LGBTQ)?*,” distinguishing respondents who identify as LGBTQ+, respondents who do not, and respondents who answered “*Preferred not to respond*” (PNTR). For parsimony, we do not present model coefficients on the PNTR category—although this category is included in the models.

Respondents’ sex recorded at birth was measured using the question “*What was your sex recorded at birth?*,” with response options being “*Male*,” “*Female*,” “*A term not listed above*” (hereon referred to as “*Other*”) and “*Preferred not to respond.*” This variable is used as a control variable in full-sample analyses separating individuals assigned male at birth (AMAB) and individuals assigned female at birth (AFAB), as well as a stratifying variable in further analyses. The latter enables us to investigate whether the wellbeing consequences of sexual-harassment victimization differ between LGBTQ+ and non-LGBTQ+ employees within each of these two sex categories.

### Other Covariates

All regression models were adjusted for an encompassing set of individual- and organization-level covariates. The specific covariates used in the analyses follow those included in earlier studies of workplace wellbeing using this and other datasets (Donaghy & Perales, 2024; Perales, 2022; Perales et al., 2022a). Collectively, they represent factors that are known to be related to LGBTQ+ status, workplace sexual harassment, and employee wellbeing, and which—if absent from the models—could plausibly result in spurious relationships between our key explanatory variables and the outcome variable through omitted-variable bias. These included: respondents’ sex recorded at birth (for pooled models only), education level, age, culturally and linguistically diverse (CALD) background, Indigenous status, disability, religiosity, neurodiversity, self-identification as a “person of color,” state of residence, contract type, job level, job tenure, and employers’ size and location. To maximize the analytic sample—and given the small degree of missing data in the predictors (up to 5.4%)—a “No information” residual category was derived and included in the model for each covariate with missing cases.

### Analytic Approach

Given the properties of the AWEI Survey data, we model employees’ wellbeing using random-intercept multilevel regression models (Goldstein, 2010). These models allow for the nesting of employees (Level 1) within organizations (Level 2), and account for organization-level unobserved effects through the inclusion of a random intercept. Formally, the models that we fit take the following form:

$$\begin{array}{l} EW_{\mathrm{io}}=\beta_{1}SH_{io}+\beta_{2}{\mathrm{LGBTQ}}_{io}+\beta_{3}(SH_{io}\times {\mathrm{LGBTQ}}_{io})\;+\beta_{4}X_{io}+\beta_{5}Z_{o}\;+\mu_{o}\\ \;\;\;\;\;\;\;\;\;\;\;\;+\epsilon_{io} \end{array}$$

where subscripts i and o stand for “individual” and “organization”; *EW* is the outcome variable capturing employee wellbeing; *SH* and *LGBTQ* are key explanatory variables capturing sexual-harassment experiences and LGBTQ+ identity; *X* and *Z* are vectors of individual- and organization-level control variables; the βs are coefficients or vectors of coefficients to be estimated; µ is an organization-level random intercept assumed to be normally distributed and orthogonal to the observables; and ε is the usual stochastic regression error term. The key parameters of interest are β_1_, β_2_, and β_3_, which together capture the interactive effect of sexual-harassment victimization and LGBTQ+ identity on employee wellbeing—β_1_ and β_2_ provide an estimate of the main effects, whereas β_3_ captures the interaction effect.

We conduct three sets of analyses. We initially fit base models of employee wellbeing that use a measure of having ever experienced sexual harassment (Table 2). We subsequently examine the role of “harassment timing” by fitting additional models using a sexual-harassment measure that separates more and less recent experiences (Table 3). For these two sets of analyses, we fit both full-sample and sex-stratified models. Finally, we estimate the effect of sexual harassment on different domains of employee wellbeing (Table 4). These analyses consist of six separate models, one for each item contributing to the workplace-wellbeing index, and use the more-nuanced sexual-harassment-victimization measure. For parsimony, the latter models are estimated on the full sample only.

## Results

### Bivariate Associations

Descriptive statistics on key analytic variables are presented in Table 1 (for other analytic variables, see Supplemental Appendix Table A1). Within the full sample, average employee wellbeing amounts to 78.51 units (on a 0–100 scale). The overall prevalence of workplace sexual-harassment experiences is 27.43%, with 2.61% of employees reporting recent (i.e., past-year) experiences and 24.82% reporting more distal experiences. Significant disparities in these key variables by LGBTQ+ status can be observed. Importantly, employee wellbeing is greater among non-LGBTQ+ (79.27 units) than LGBTQ+ respondents (76.66 units). In contrast, LGBTQ+ respondents exhibit greater exposure to workplace sexual harassment: 35.16% compared to 23.31% for having ever experienced sexual harassment, 5.16% compared to 1.83% for recent sexual harassment, and 30.00% compared to 23.31% for more distal sexual harassment. Results from *t*-tests and ANOVA tests confirmed that these differences are all statistically significant (p < .001 in all cases).

**Table 1.** Descriptive Statistics for Key Analytic Variables, by LGBTQ+ Status.

	Full Sample		LGBTQ+ Respondents		Non-LGBTQ+ Respondents	
Variables	Mean/%	*SD*	Mean/%	*SD*	Mean/%	*SD*
Employee wellbeing, mean						
Composite index (range: 0–100)	78.51	19.23	76.66	20.56	79.27	18.61
Employee wellbeing: Safety and inclusion (range: 1–5)	4.42	0.78	4.40	0.82	4.43	0.75
Employee wellbeing: Mental wellbeing (range: 1–5)	4.01	0.98	3.88	1.07	4.05	0.94
Employee wellbeing: Being oneself (range: 1–5)	4.12	0.95	4.00	1.06	4.16	0.90
Employee wellbeing: Productivity (range: 1–5)	4.22	0.80	4.18	0.85	4.23	0.77
Employee wellbeing: Engagement (range: 1–5)	4.07	0.94	4.00	1.01	4.10	0.91
Employee wellbeing: Sense of belonging (range: 1–5)	4.02	0.98	3.93	1.05	4.05	0.95
Sexual harassment at work, %						
No, never	72.57		64.84		74.86	
Yes, within the past 12 months	2.61		5.16		1.83	
Yes, more than 12 months ago	24.82		30.00		23.31	
Ever experienced sexual harassment at work, %	27.43		35.16		25.14	
Sex recorded at birth, %						
Male	41.14		46.98		39.58	
Female	58.13		52.19		60.19	
Other	0.07		0.19		0.02	
PNTR	0.66		0.65		0.21	
LGBTQ+ identity, %						
Not LGBTQ+	76.08		0.00		100.00	
LGBTQ+	22.22		100.00		0.00	
PNTR	1.70		0.00		0.00	

*Note. SD* = standard deviation; LGBTQ+ = lesbian, gay, bisexual, trans, and queer; PNTR = preferred not to respond.

*Source*. 2022 Australian Workplace Equality Index Employee Survey.

### Estimated Effects of Sexual Harassment on Employee Wellbeing

Table 2 presents the results of a base set of regression models of employee wellbeing using a measure of having ever experienced sexual harassment (selected measures of effect magnitude and reconstituted interaction effects are presented in Supplemental Appendix Table A2). The coefficients on the sexual-harassment main effect indicate that—among non-LGBTQ+ employees—having experienced sexual harassment is associated with negative and statistically significant decreases in employee wellbeing.^2^ On a 0–100 scale, these decreases amount to 4.45 units for the full sample (p < .001), 6.82 units for the AMAB sample (p < .001), and 3.99 units for the AFAB sample (p < .001). The coefficients on the interaction terms give the difference in the estimated effects of sexual harassment on employee wellbeing between non-LGBTQ+ and LGBTQ+ respondents. These coefficients are negative in all three cases, pointing to larger “penalties” among LGBTQ+ individuals. However, only the coefficient for the full-sample model achieves statistical significance (β = −2.18; p < .001). This indicates that, within the pooled sample, the deleterious consequences of sexual-harassment victimization on employee wellbeing are felt more strongly by LGBTQ+ employees. Specifically, in the full-sample model, the predicted decrease in employee wellbeing associated with having ever experienced sexual harassment is 4.45 units for non-LGBTQ+ employees, compared to 6.63 units (i.e., −4.45 + [−2.18] = −6.63, see Supplemental Appendix Table A2) for LGBTQ+ employees.^3^

**Table 2.** Random-Intercept Multilevel Regression Models of Employee Wellbeing (Range: 0–100), Effects of Having Ever Experienced Sexual Harassment.

Variables	All	AMAB	AFAB
Ever experienced sexual harassment	−4.45***	−6.82***	−3.99***
LGBTQ+	0.25	1.90***	−1.91***
LGBTQ+ × Ever experienced sexual harassment	−2.18***	−1.56	−0.35
*N* (observations)	38,280	15,751	22,251
*N* (organizations)	179	177	178
Overall *R*^2^	.08	.09	.07

*Note*. Models are adjusted for respondents’ sex recorded at birth (full-sample-model only), education level, age group, culturally and linguistically diverse status, Indigenous status, disability status, religiosity, neurodiversity, self-identification as a person of color, state of residence, area remoteness, contract type, job level, job tenure, and employers’ sector and size. LGBTQ+ = lesbian, gay, bisexual, trans, and queer; AMAB = assigned male at birth; AFAB = assigned female at birth.

*Source*. 2022 Australian Workplace Equality Index Employee Survey.

Statistical significance: *p < .05. **p < .01. *** *p* < .001.

### Timing of Sexual Harassment

Table 3 presents the results of analogous models of employee wellbeing comparing the effects of more and less recent sexual-harassment experiences. The models reveal large decreases in employee wellbeing associated with more recent—compared to less recent—sexual-harassment experiences. In the non-LGBTQ+ subsample, recent harassment experiences decrease employee wellbeing by −13.16 units (p < .001) overall, −14.56 units (p < .001) for AMAB people, and −11.94 units (p < .001) for AFAB people. In comparison, decreases stemming from more distal sexual-harassment experiences amount to −3.73 units (p < .001) overall, −5.63 units (p < .001) for AMAB people, and −3.45 units (p < .001) for AFAB people. The models reveal no differences by LGBTQ+ identity in the negative impacts of distal sexual-harassment experiences, as denoted by non-statistically-significant coefficients on the interaction terms (p > .05). The pattern of results is, however, different for recent sexual-harassment experiences. These experiences exert a larger negative effect on LGBTQ+ individuals in the full (β = −4.83; p < .001) and AMAB (β = −9.94; p < .001) samples. In the full sample, recent harassment experiences reduce employee wellbeing by 13.16 units among non-LGBTQ+ people, compared to 17.99 units among LGBTQ+ people (i.e., −13.16 + [−4.83] = −17.99, Supplemental Appendix Table A2). In the AMAB sample, the analogous decreases amount to 14.56 and 24.5 units (i.e., −14.56 + [−9.94] = 24.50, Supplemental Appendix Table A2), respectively.

**Table 3.** Random-Intercept Multilevel Regression Models of Employee Wellbeing (Range: 0–100), Timing of Sexual Harassment.

Variables	All	AMAB	AFAB
Sexual-harassment experiences (Ref. No, never)			
Yes, more than 12 months ago	−3.73***	−5.63***	−3.45***
Yes, within the past 12 months	−13.16***	−14.56***	−11.94***
LGBTQ+	0.18	1.89***	−2.03***
Interactions			
LGBTQ+ × Yes, more than 12 months ago	−0.94	0.11	0.48
LGBTQ+ × Yes, within the past 12 months	−4.83***	−9.94***	−0.48
*N* (observations)	38,280	15,751	22,251
*N* (organizations)	179	177	178
Overall *R*^2^	.09	.10	.07

*Note*. Models are adjusted for respondents’ sex recorded at birth (full-sample-model only), education level, age group, culturally and linguistically diverse status, Indigenous status, disability status, religiosity, neurodiversity, self-identification as a person of color, state of residence, area remoteness, contract type, job level, job tenure, and employers’ sector and size. LGBTQ+ = lesbian, gay, bisexual, trans, and queer; AMAB = assigned male at birth; AFAB = assigned female at birth.

*Source*. 2022 Australian Workplace Equality Index Employee Survey.

Statistical significance: *p < .05. **p < .01. ***p < .001.

### Domains of Employee Wellbeing

Table 4 presents the results of a final set of models that separately consider the estimated effects of sexual-harassment experiences on the different items contributing to the workplace-wellbeing index (each measured on a 1–5 scale). The results reveal some interesting patterns. First, for the non-LGBTQ+ subsample, both recent and distal sexual-harassment-victimization experiences are associated with decreases across all workplace-wellbeing domains. These decreases range from −0.30 units (“productivity”) to −0.66 units (“mental wellbeing at work”) for recent experiences, and from −0.10 (“safety and inclusion”) to −0.19 units (“being oneself at work” & “mental wellbeing at work”) for more distal experiences. Second, across domains, additional “penalties” are observed for LGBTQ+ compared to non-LGBTQ+ employees, as denoted by statistically significant coefficients in 8 of 12 interaction terms. Disparities are most noticeable for recent sexual-harassment experiences (six of six interaction terms are significant) compared to more distal experiences (two of six terms). Disparities in the health-and-wellbeing impacts of recent sexual-harassment experiences between LGBTQ+ and other employees are most pronounced for items pertaining to “being oneself at work” (−0.27 units), “safety and inclusion,” (−0.24 units) and “sense of belonging” (−0.23 units).

**Table 4.** Random-Intercept Multilevel Regression Models of Employee Wellbeing (Full Sample), Specific Domains (Range: 1–5).

Variables	Item 1	Item 2	Item 3	Item 4	Item 5	Item 6
	Safety & Inclusion	Mental Wellbeing	Being Oneself	Productivity	Engagement	Sense of Belonging
Sexual-harassment experiences (Ref. No never)						
Yes, more than 12 months ago	−.10*	−.19*	−.19*	−.12*	−.14*	−.16*
Yes, within the past 12 months	−.53*	−.66*	−.58*	−.30*	−.50*	−.55*
LGBTQ+	.04*	−.04**	–.05**	.04**	.03	.00
Interactions						
LGBTQ+ × Yes, more than 12 months ago	−.04*	−.03	−.04	−.00	−.06*	−.04
LGBTQ+ × Yes, within the past 12 months	−.23*	−.12*	−.27*	−.17*	−.14*	−.24*
*N* (observations)	38,267	38,257	38,253	38,250	38,245	38,239
*N* (organizations)	179	179	179	179	179	179
Overall *R*^2^	.07	.08	.08	.04	.07	.07

*Note*. Models are adjusted for respondents’ sex, education level, age group, culturally and linguistically diverse status, Indigenous status, disability status, religiosity, neurodiversity, self-identification as a person of color, state of residence, area remoteness, contract type, job level, job tenure, and employers’ sector and size. LGBTQ+ = lesbian, gay, bisexual, trans, and queer.

*Source*. 2022 Australian Workplace Equality Index Employee Survey.

Statistical significance: *p < .05. **p < .01. *p < .001.

## Discussion and Conclusion

Despite widespread recognition of the individual and societal risks associated with workplace sexual harassment in academic, media, and policy circles, few studies have considered how the detrimental health-and-wellbeing impacts of victimization differ across population groups. The present study has offered novel evidence pertaining to an important and under-researched subpopulation—namely, individuals who identify as LGBTQ+. To accomplish this, we leveraged rich and unique data from the 2022 AWEI Employee Survey. Consistent with results from studies in other countries (Blindow et al., 2021; Blindow et al., 2022, 2023; Magnusson Hanson et al., 2020; Rugulies et al., 2020; Sojo et al., 2016; Sterud et al., 2023), our Australian analyses revealed large and statistically significant decreases in workplace-wellbeing stemming from workplace-sexual-harassment victimization. The magnitude of these estimated effects was visibly large. For example, for non-LGBTQ+ individuals of either sex, it amounted to 23% of a standard deviation (*SD*) for any experiences of sexual harassment, 68% for past-year harassment, and 19% for more distal harassment.

Critically, and consistent with our main hypothesis, the results also provided novel evidence that workplace sexual harassment exerts significantly more detrimental effects on LGBTQ+ than non-LGBTQ+ employees, *ceteris paribus*. Within the full LGBTQ+ employee sample, sexual-harassment victimization (experienced at any point) led to wellbeing decreases amounting to 35% of an *SD* (or 11 percentage points more than for non-LGBTQ+ respondents). Similar disparities were observed for recent sexual-harassment exposure within the full sample (94% of an *SD*, 25 additional percentage points) and the AMAB sample (120% of an *SD*, 48 additional percentage points). While our analyses do not elucidate the specific mechanisms contributing to the reported patterns of association, the results are highly consistent with stigma-based perspectives on LGBTQ+ disadvantage, including the minority stress model (Meyer, 2003; Perales & Todd, 2018), and with theoretical tenets from the stress process model (Pearlin, 1989, 2010; Pearlin et al., 1981). These perspectives underscore how unique stressors at the micro, meso, and macro levels pollute the lives of LGBTQ+ individuals, resulting in comparatively higher levels of accumulated stress. These factors, we argued, compound with sexual-harassment experiences to yield larger health-and-wellbeing penalties among LGBTQ+ employees.

In addition, and consistent with the stress process (Pearlin, 1989, 2010; Pearlin et al., 1981), our analyses revealed that recent harassment experiences are more strongly tied to poor employee wellbeing than distal harassment experiences—particularly for LGBTQ+ individuals. This finding signals that recovery from the trauma caused by sexual-harassment victimization takes time. Greater challenges for LGBTQ+ employees to “flee” organizations where harassment occurred (Dilmaghani & Robinson, 2024; Mills & Oswin, 2024), at least within the short run, may explain why they are disproportionately affected by recent victimization experiences—but not by more distal ones. The models further unveiled that the impacts of workplace sexual harassment extend to all measured domains of employee wellbeing (Lyubomirsky, 2001; Page & Vella-Brodrick, 2009; Wijngaards et al., 2022; Zheng et al., 2015). This evidences that the stress and trauma induced by sexual-harassment victimization are multidimensional in nature, permeating multiple aspects of employee’s workplace experiences. Interestingly, differences in the overall impacts of harassment on the wellbeing of LGBTQ+ and non-LGBTQ+ employees were driven by larger LGBTQ+ penalties on domains pertaining to “being oneself at work,” “safety and inclusion,” and “sense of belonging.” This is consistent with the earlier proposition that workplace sexual harassment against LGBTQ+ employees specifically targets their sexual and gender identities in an attempt to invalidate or “other” them (TUC, 2019), resulting in more profound health-and-wellbeing consequences.

Overall, the current study featured multiple strengths that enabled us to generate new knowledge on several under-researched analytic pathways. For example, our analyses leveraged data from a unique employer-employee dataset comprising rich information on LGBTQ+ identity, sexual-harassment experiences, and employee wellbeing; relied on an unusually large sample of LGBTQ+ respondents (n = 9,806); and provided new insights into heterogeneity in the intra-individual impacts of sexual-harassment victimization (by LGBTQ+ status, harassment timing, and wellbeing domain). Despite this, certain study limitations must be borne in mind. For instance, the available data did not enable unpacking how different manifestations of sexual harassment (e.g., in terms of their nature, recurrence, or intensity) influence wellbeing. In addition, the AWEI Survey relies on a non-probability sampling approach, which precludes direct generalizations to the population it represents and calls for caution in interpreting inferential statistics. Our study thus invites—and hopefully paves the way for—future research that both refines and expands on our findings. Of particular importance are new studies that: address the mechanisms underpinning the identified associations; unpack heterogeneity within the LGBTQ+ umbrella (including differences between gender diversity and sexuality diversity); consider how LGBTQ+ identity intersects with other disadvantaged social locations (e.g., ethnic-minority status or disability); rely on probability-based surveys (as new, suitable data sources become available); and unpack the potentially disparate health-and-wellbeing effects of diverse manifestations of workplace sexual harassment (e.g., LGBTQ-targeted vs. generic instances of harassment). Some of these research avenues could be pursued by in-depth qualitative studies that incorporate victims’ first-hand accounts. Indeed, the value of qualitative research in the study of violence is well recognized—see Hardesty et al. (2019) for an encompassing articulation, and Scarduzio et al. (2018) and Taylor-Dunn et al. (2021) for recent empirical examples.

Despite the opportunity for future refinement, our findings bear significant lessons for organizational policy and practice. Overall, given the large wellbeing penalties observed, our results underscore the urgency and critical importance of preventive and remedial actions aimed at mitigating workplace sexual harassment. At the same time, the patterns observed for LGBTQ+ employees call for concentrated efforts and targeted responses addressing this subpopulation. For the first time, this study demonstrates that LGBTQ+ employees suffer a “double burden” in relation to workplace-sexual-harassment victimization: they are not only more likely to be exposed to it, but also experience more profound decreases in wellbeing when it occurs. As a result, sexual harassment may act as a vehicle to reinforce entrenched social hierarchies within the workplace, actively contributing to the socioeconomic exclusion of LGBTQ+ employees (Konik & Cortina, 2008; Perales et al., 2024). Targeted solutions to prevent this situation are urgently required. For instance, ensuring that employees with diverse genders and sexualities are appropriately represented at all levels of the organizational hierarchy—and thus play a role in designing policies and overseeing grievance procedures—has been argued to be key to preventing and appropriately responding to workplace sexual harassment (Clarke, 2020; Ellsworth et al., 2020; García Johnson & Otto, 2019). Overall, our findings make it clear that efforts to mitigate workplace sexual harassment should move hand-in-hand with diversity and inclusion policies that make workplaces a safe environment for individuals of all genders and sexualities.

## Supplemental Material

sj-docx-1-jiv-10.1177_08862605241285994 – Supplemental material for Workplace-Sexual-Harassment Victimization and Employee Wellbeing Among LGBTQ+ and Non-LGBTQ+ Employees Supplemental material, sj-docx-1-jiv-10.1177_08862605241285994 for Workplace-Sexual-Harassment Victimization and Employee Wellbeing Among LGBTQ+ and Non-LGBTQ+ Employees by Francisco Perales, Alice Campbell and Nicki Elkin in Journal of Interpersonal Violence

## Notes

1. Given the sensitive nature of sexual harassment, as for other survey datasets, there may be a degree of underreporting of workplace-sexual-harassment victimization within the AWEI Employee Survey data. For example, some victims may not interpret certain in-scope behaviors as falling under the definition of sexual harassment, or prefer not to disclose their experiences in the context of a workplace survey (see e.g., Charlesworth et al., 2011; Shupe, 2020).

2. Despite the richness of the explanatory variables included in the regression models, the associated *R*^2^ statistics are modest: .07 for the AFAB model, .08 for the pooled model, and .09 for the AMAB model. This underscores the complexity of employee wellbeing, which represents a highly idiosyncratic and multifaceted construct that is difficult to predict using conventional survey questions.

3. While not a key focus of the present study, our analyses also reveal some statistically significant relationships between the control variables and employee wellbeing (see Supplemental Appendix Table A3). These associations are consistent with those reported in earlier studies of workplace wellbeing (see e.g., Donaghy & Perales, 2024; Perales, 2022; Perales et al., 2022a). For example, we observe associations between higher employee wellbeing and factors such as being AFAB, living in a metropolitan area, working standard full-time hours, having a higher position within the organization, working for the private sector, working for a small organization, and having recently joined one’s employer. In contrast, having a CALD background, being Indigenous, having a disability, being neurodiverse, and identifying as a “person of color” were factors associated with lower employee wellbeing. There were also significant employee-wellbeing differences across states within Australia, but few patterned effects for age group or education level.
