# Supplementary material for: Workplace-Sexual-Harassment Victimization and Employee Wellbeing Among LGBTQ+ and Non-LGBTQ+ Employees
Source: J Interpers Violence. 2024 Oct 8;40(17-18):4203–25. doi: 10.1177/08862605241285994 (PMC12308041; doi:10.1177/08862605241285994)
Supplement: sj-docx-1-jiv-10.1177_08862605241285994 – Supplemental material for Workplace-Sexual-Harassment Victimization and Employee Wellbeing Among LGBTQ+ and Non-LGBTQ+ Employees [file sj-docx-1-jiv-10.1177_08862605241285994.docx]

**Online Appendix**

Table A1. Descriptive statistics on other analytic variables (%s)

|  | Full sample | LGBTQ+ respondents | Non-LGBTQ+ respondents |
| --- | --- | --- | --- |
| Education level |  |  |  |
| Degree or higher | 67.37 | 65.13 | 68.03 |
| Certificate or diploma | 21.14 | 21.46 | 21.05 |
| Lower or other qualification | 11.50 | 13.41 | 10.92 |
| Age group |  |  |  |
| <34 years | 29.38 | 46.43 | 24.44 |
| 35-54 years | 55.48 | 46.12 | 58.31 |
| 55+ years | 14.10 | 7.01 | 16.21 |
| PNTR | 1.04 | 0.45 | 1.04 |
| CALD background |  |  |  |
| No | 85.92 | 83.05 | 86.88 |
| Yes | 9.69 | 9.02 | 9.83 |
| No info | 4.39 | 7.93 | 3.28 |
| Indigenous status |  |  |  |
| No | 93.55 | 88.98 | 94.99 |
| Yes | 2.06 | 3.09 | 1.73 |
| No information | 4.39 | 7.93 | 3.28 |
| Disability |  |  |  |
| No | 90.88 | 84.31 | 92.92 |
| Yes | 4.74 | 7.76 | 3.79 |
| No information | 4.39 | 7.93 | 3.28 |
| Religious/person of faith |  |  |  |
| No | 84.49 | 86.37 | 84.16 |
| Yes | 11.12 | 5.70 | 12.55 |
| No information | 4.39 | 7.93 | 3.28 |
| Neurodiversity |  |  |  |
| No | 89.93 | 78.35 | 93.45 |
| Yes | 5.68 | 13.72 | 3.27 |
| No information | 4.39 | 7.93 | 3.28 |
| Self-identification as a ‘person of color’ |  |  |  |
| No | 86.88 | 83.73 | 87.98 |
| Yes | 8.73 | 8.33 | 8.74 |
| No information | 4.39 | 7.93 | 3.28 |
| State of residence |  |  |  |
| New South Wales | 31.06 | 30.09 | 31.31 |
| Victoria | 24.00 | 23.10 | 24.27 |
| Queensland | 15.16 | 15.50 | 15.11 |
| Australian Capital Territory | 11.74 | 14.39 | 10.95 |
| Western Australia | 12.07 | 10.75 | 12.45 |
| South Australia | 4.38 | 4.42 | 4.37 |
| Tasmania | 0.95 | 1.01 | 0.92 |
| Northern Territory | 0.64 | 0.74 | 0.60 |
| Area remoteness |  |  |  |
| City/Metropolitan | 82.89 | 84.51 | 82.49 |
| Regional/Rural | 13.22 | 12.12 | 13.53 |
| Remote | 3.90 | 3.37 | 3.98 |
| Contract type |  |  |  |
| Full-time | 83.53 | 83.26 | 83.59 |
| Part-time | 9.85 | 7.53 | 10.56 |
| Fixed-term | 4.20 | 5.16 | 3.91 |
| Temporary/casual | 2.14 | 3.73 | 1.69 |
| Another employment type | 0.28 | 0.32 | 0.25 |
| Job tenure |  |  |  |
| Less than 1 year | 20.08 | 24.82 | 18.74 |
| 1-3 years | 19.33 | 23.14 | 18.18 |
| 3-5 years | 13.33 | 14.08 | 13.14 |
| More than 5 years | 47.26 | 37.95 | 49.94 |
| Job level |  |  |  |
| Senior/executive management | 8.61 | 5.55 | 9.59 |
| Middle management | 35.08 | 31.46 | 36.17 |
| Regular employee | 47.16 | 51.77 | 45.73 |
| Other | 9.15 | 11.21 | 8.51 |
| Employment sector |  |  |  |
| Private Sector | 53.93 | 48.44 | 55.54 |
| Public Sector | 36.82 | 40.35 | 35.77 |
| Not-for-Profit | 2.75 | 2.30 | 2.88 |
| Higher Education | 6.51 | 8.91 | 5.80 |
| Employer size |  |  |  |
| Small (<500 employees) | 4.55 | 3.41 | 4.90 |
| Medium (501-1,999 employees) | 20.06 | 16.42 | 21.14 |
| Large (2,000-8,000 employees) | 43.13 | 42.62 | 43.24 |
| Significant (>8,000 employees) | 32.25 | 37.55 | 30.72 |

Notes: 2022 Australian Workplace Equality Index Employee Survey. LGBTQ+: Lesbian, Gay, Bisexual, Trans or Queer. PNTR: Prefer Not to Respond. CALD: Culturally and Linguistically Diverse.

Table A2. Magnitude of the estimated effects on the sexual-harassment explanatory variables

|  | Model parameters | | | % of a SD in employee  wellbeing (Cohen’s d) | | |
| --- | --- | --- | --- | --- | --- | --- |
|  | All | Male | Female | All | Male | Female |
| SD for employee wellbeing (Range: 0 to 100) | 19.23 | 20.56 | 18.61 | – | – | – |
| Estimated effects of having ever experienced sexual harassment |  |  |  |  |  |  |
| Non-LGBTQ+ individuals | –4.48 | –6.82 | –3.99 | –23% | –33% | –21% |
| LGBTQ+ individuals | –6.66 | –8.38 | –4.34 | –35% | –41% | –23% |
| Group difference | –2.18 | –1.56 | –0.35 | –11% | –8% | –2% |
| Estimated effects of sexual harassment over 12 months ago |  |  |  |  |  |  |
| Non-LGBTQ+ individuals | –3.73 | –5.63 | –3.45 | –19% | –27% | –19% |
| LGBTQ+ individuals | –4.67 | –5.52 | –2.97 | –24% | –27% | –16% |
| Group difference | –0.94 | 0.11 | 0.48 | –5% | 1% | 3% |
| Estimated effects of sexual harassment within past 12 months |  |  |  |  |  |  |
| Non-LGBTQ+ individuals | –13.16 | –14.56 | –11.94 | –68% | –71% | –64% |
| LGBTQ+ individuals | –17.99 | –24.50 | –12.42 | –94% | –119% | –67% |
| Group difference | –4.83 | –9.94 | –0.48 | –25% | –48% | –3% |

Notes: 2022 Australian Workplace Equality Index Employee Survey. SD: Standard deviation. LGBTQ+: Lesbian, Gay, Bisexual, Trans or Queer. Results based on the regression models presented in Tables 2 and 3. Grey-shaded cells are based on model parameters that are not statistically significant.

Table A3. Random-intercept multilevel regression models of employee wellbeing (range: 0 to 100), effects of having ever experienced sexual harassment (full set of regression results)

|  | All | AMAB | AFAB |
| --- | --- | --- | --- |
| Ever experienced sexual harassment | –4.48^***^ | –6.82^***^ | –3.99^***^ |
| LGBTQ+ | 0.25 | 1.90^***^ | –1.91^***^ |
| LGBTQ+ × Ever experienced sexual harassment | –2.18^***^ | –1.56 | –0.35 |
| Sex *(reference category)* |  |  |  |
| AMAB |  |  |  |
| AFAB | 3.01^***^ |  |  |
| A different term | –5.11 |  |  |
| Education level |  |  |  |
| Degree or higher *(reference category)* |  |  |  |
| Certificate or diploma | –0.20 | –1.43^***^ | 0.78^*^ |
| Lower or other qualification | 0.28 | 0.20 | 0.38 |
| Age group |  |  |  |
| <34 years *(reference category)* |  |  |  |
| 35-54 years | 0.20 | –0.52 | 0.55 |
| 55+ years | 0.29 | –0.13 | 0.47 |
| CALD background | –0.81^*^ | –0.44 | –1.04^*^ |
| Indigenous | –3.41^***^ | –2.23^*^ | –3.66^***^ |
| Has a disability | –4.15^***^ | –4.97^***^ | –3.66^***^ |
| Religious/person of faith | 0.20 | 0.91 | –0.28 |
| Has a neurodiversity | –5.60^***^ | –6.36^***^ | –5.07^***^ |
| Self-identification as a ‘person of color’ | –0.89^*^ | –0.26 | –1.22^**^ |
| State of residence |  |  |  |
| New South Wales *(reference category)* |  |  |  |
| Victoria | –0.15 | –0.52 | –0.07 |
| Queensland | –0.66^*^ | –0.53 | –0.74 |
| Australian Capital Territory | –0.97^**^ | –1.52^**^ | –0.03 |
| Western Australia | –2.62^***^ | –2.33^***^ | –1.92^***^ |
| South Australia | –1.24^*^ | –1.17 | –1.01 |
| Tasmania | 0.05 | –1.70 | 0.42 |
| Northern Territory | –1.01 | 0.17 | –1.63 |
| Area remoteness |  |  |  |
| City/Metropolitan *(reference category)* |  |  |  |
| Regional/Rural | –1.53^***^ | –2.35^***^ | –1.10^**^ |
| Remote | –2.30^***^ | –1.35 | –2.83^***^ |
| Contract type |  |  |  |
| Full-time *(reference category)* |  |  |  |
| Part-time | –1.14^***^ | –1.61 | –1.24^***^ |
| Fixed-term | 0.49 | 0.31 | 0.53 |
| Temporary/casual | –2.05^**^ | –2.92^*^ | –1.22 |
| Another employment type | –2.71 | –2.09 | –2.16 |
| Job tenure |  |  |  |
| Less than 1 year *(reference category)* |  |  |  |
| 1-3 years | –3.51^***^ | –3.51^***^ | –3.50^***^ |
| 3-5 years | –4.31^***^ | –4.28^***^ | –4.45^***^ |
| More than 5 years | –4.82^***^ | –5.81^***^ | –4.19^***^ |
| Job level |  |  |  |
| Senior/executive management *(reference category)* |  |  |  |
| Middle management | –4.98^***^ | –5.38^***^ | –4.63^***^ |
| Regular employee | –7.65^***^ | –8.14^***^ | –7.22^***^ |
| Other | –7.40^***^ | –7.65^***^ | –7.25^***^ |
| Employment sector |  |  |  |
| Private Sector *(reference category)* |  |  |  |
| Public Sector | –2.91^***^ | –2.55^***^ | –3.53^***^ |
| Not-for-Profit | 1.83^**^ | 3.67^***^ | 0.44 |
| Higher Education | –3.14^***^ | –2.71^***^ | –3.54^***^ |
| Employer size |  |  |  |
| Small (<500 employees) *(reference category)* |  |  |  |
| Medium (501-1,999 employees) | –1.44^**^ | –2.28^**^ | –0.76 |
| Large (2,000-8,000 employees) | –2.40^***^ | –3.71^***^ | –1.37 |
| Significant (>8,000 employees) | –3.87^***^ | –4.59^***^ | –3.36^***^ |
| N (observations) | 38,280 | 15,751 | 22,251 |
| N (organizations) | 179 | 177 | 178 |
| Overall R^2^ | 0.08 | 0.09 | 0.07 |

Notes: 2022 Australian Workplace Equality Index Employee Survey. LGBTQ+: Lesbian, Gay, Bisexual, Trans or Queer. AMAB: Assigned male at birth. AMAF: Assigned female at birth. For parsimony, model coefficients on the ‘Prefer not to respond’ and ‘Missing information’ residual dummy variables are not reported. Statistical significance: ^*^ *p*<0.05, ^**^ *p*<0.01, ^***^ *p*<0.001.
